# Changes of fundus autofluorescence and spectral-domain optical coherence tomographic findings after treatment of primary intraocular lymphoma

**DOI:** 10.1186/1869-5760-4-7

**Published:** 2014-02-22

**Authors:** Mariko Egawa, Yoshinori Mitamura, Yuki Hayashi, Kentaro Semba, Takeshi Naito

**Affiliations:** 1 Department of Ophthalmology, Institute of Health Biosciences, The University of Tokushima Graduate School, 3-18-15 Kuramoto, Tokushima 770-8503, Japan

**Keywords:** Electroretinograms, Fundus autofluorescence, Humphrey visual field test, Microperimetry, Multifocal electroretinograms, Primary intraocular lymphoma, Spectral-domain optical coherence tomography

## Abstract

**Background:**

We report the fundus autofluorescence (FAF), spectral-domain optical coherence tomographic (SD-OCT), microperimetric, and multifocal electroretinographic (mfERG) findings before, during, and after successful treatment of a primary intraocular lymphoma (PIOL).

**Findings:**

A 57-year-old man had biopsy-proven PIOL in his left eye, and he was treated with intravitreal methotrexate injections for 8 months. Before treatment, fundus examination disclosed many small, yellow lesions with distinct boundaries in the posterior fundus which became atrophic 9 months after the initial treatment. FAF showed a pattern of granular hypoautofluorescence and hyperautofluorescence before the treatments and patchy hypoautofluorescence corresponding to retinal pigment epithelial (RPE) atrophy after the treatments. SD-OCT showed increased nodularity at the level of and above the RPE, a separation of Bruch membrane from the RPE, partial damage of the RPE, disruption of the photoreceptor inner segment/outer segment (IS/OS) junction, multiple hyperreflective signals in the inner retina, foveal thinning, and parafoveal thickening. After treatment, the hyperreflective infiltrations in the inner retina were markedly decreased, and the RPE and IS/OS junction were restored. The foveal thinning and parafoveal thickening resolved, and the central choroidal thickness decreased. During the follow-up, the mfERGs remained decreased. In contrast, microperimetry showed a partial improvement of the retinal sensitivity.

**Conclusion:**

FAF and SD-OCT are useful noninvasive methods to evaluate the retinal and choroidal changes before and after treatment of PIOL. Our results suggest that visual recovery after successful treatment may be limited once macula is infiltrated.

## Findings

### Introduction

A primary intraocular lymphoma (PIOL) is a type of primary central nervous system lymphoma (PCNSL). Patients with a PIOL often have iritis, vitreous opacities, and retinal infiltrations and are often misdiagnosed as having uveitis. Fundus autofluorescence (FAF) imaging has been recently used to diagnose various retinal diseases such as retinitis pigmentosa [1,2]. The autofluorescence pattern in the FAF images depends on the distribution of lipofuscin in the retinal pigment epithelial (RPE) cells. An increase in the hyperautofluorescence indicates an increase in the lipofuscin in the RPE due to the degeneration of the photoreceptor outer segments [3].

Several studies have reported on the FAF findings such as the granular pattern in the eyes with a PIOL [4,5]. We have also reported on the FAF images in two cases of PIOL before treatment [6]. To the best of our knowledge, there is only one report describing changes of the FAF findings in a case of PIOL after successful treatment [4].

The purpose of this study was to follow the longitudinal changes of the FAF images, spectral-domain optical coherence tomographic (SD-OCT) images, retinal sensitivities determined by microperimetry, and multifocal electroretinograms (mfERGs) after a successful treatment of an eye with a PIOL. An approval was obtained from the Institutional Review Board of Tokushima University Hospital to perform this study. This study is in compliance with the Helsinki Declaration. Also, the patient has given consent for the report to be published.

### Case report

A 57-year-old man presented with decreased vision in the left eye of 1-month duration. At the initial examination, the best-corrected visual acuity (BCVA) was 20/25. Ophthalmoscopy showed retinal infiltrations in the posterior pole and diffuse vitreous opacities. Diagnostic vitrectomy was performed, and the cytodiagnosis revealed class V atypical lymphocytes. The interleukin (IL)-10 level was 3,150 pg/ml, IL-6 level was 102 pg/ml, and the IL-10/IL-6 ratio of the vitreous was 30.9 which led to a diagnosis of PIOL. Because a PCNSL was not detected by magnetic resonance imaging, the patient was treated with intravitreal injections of methotrexate (MTX). After weekly intravitreal MTX injections (400 μg/0.1 ml) for 1 month, the level of IL-10 in the aqueous humor became undetectable, and then monthly maintenance injections were performed for seven more months.

**Figure 1:**
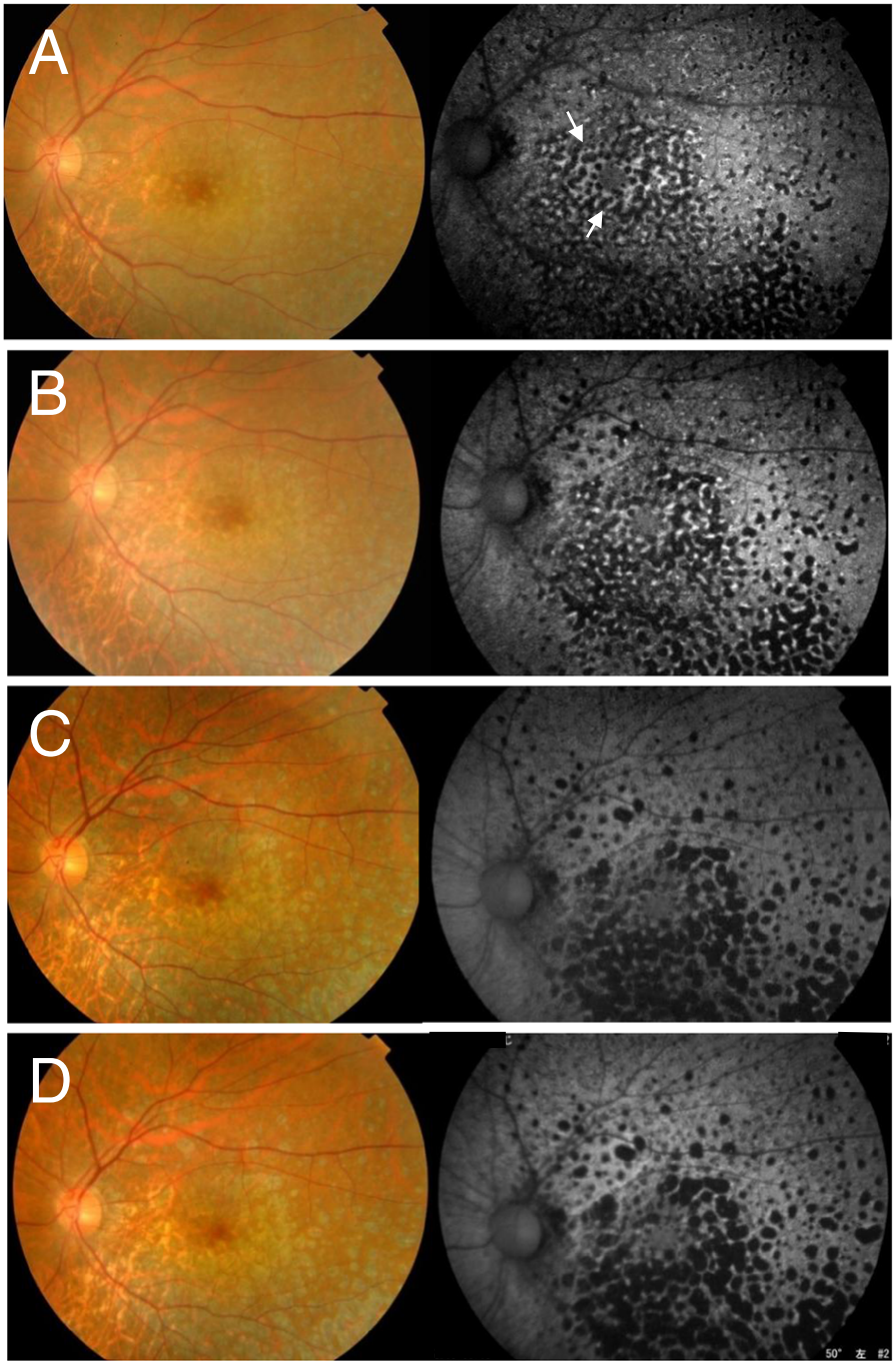
**Fundus and fundus autofluorescence (FAF) findings in the eye with primary intraocular lymphoma (PIOL). (A)** Before treatment. Fundus photograph (left) shows small yellowish lesions resembling drusen in the posterior fundus, and FAF (right) shows a granular pattern of slight hypoautofluorescence and hyperautofluorescent rings (arrows). **(B)** One month after the initial methotrexate injection. **(C)** Six months after the initial injection. **(D)** Nine months after the initial injection. The yellowish lesions gradually change to retinal pigment epithelial (RPE) atrophy in the fundus photographs, and the patchy hypoautofluorescence corresponding to the RPE atrophy is seen in the FAF images 9 months after the initial injection.

We evaluated the FAF and SD-OCT images, visual fields, microperimetric, mfERG, and full-field ERG findings before and 1, 6, and 9 months after the initial MTX injection. The BCVA was 20/20 before treatment and improved to 20/16 after 9 months (Table 1). Before treatment, fundus examination showed many small, yellowish lesions with distinct boundaries resembling drusen in the posterior fundus (Figure 1A, left). After treatment, the yellowish lesions gradually changed to atrophy of the RPE (Figure 1B,C,D, left).

**Table 1:** Chronological change of ophthalmologic findings in the left eye with PIOL after treatment

	**Before treatment**	**After treatment**		
		**1 M**	**6 M**	**9 M**
BCVA	20/20	20/25	20/16	20/16
Mean deviation on HFA (dB)	-13.95	N/A	N/A	-7.23
Mean retinal sensitivity on microperimetry (dB)	0.3	1.25	2.9	4.1
Full-field ERG	Subnormal	N/A	N/A	Subnormal
Amplitudes of mfERG	Markedly decreased	Markedly decreased	Markedly decreased	Markedly decreased
Total macular volume (6.0-mm circle, mm^3^)	10.2	8.04	8.17	8.5
Foveal retinal thickness (μm)	155	178	198	195
Subfoveal choroidal thickness (μm)	245	201	170	170
Length of IS/OS junction (μm)	Not detectable	453	849	904

BCVA, best-corrected visual acuity; ERG, electroretinogram; IS/OS, inner segment/outer segment; HFA, Humphrey Field Analyzer; M, months; mfERG, multifocal electroretinogram; N/A not available.

FAF was performed with the Topcon TRC-50DX retinal camera (Topcon, Tokyo, Japan) with an excitation bandpass filter of 535 to 585 nm and a barrier bandpass filter of 615 to 715 nm. FAF showed a slight hypoautofluorescence and hyperautofluorescence granular pattern before treatment (Figure 1A, right), and these lesions changed to patchy hypoautofluorescence corresponding RPE atrophy after the treatments (Figure 1B,C,D, right).

**Figure 2:**
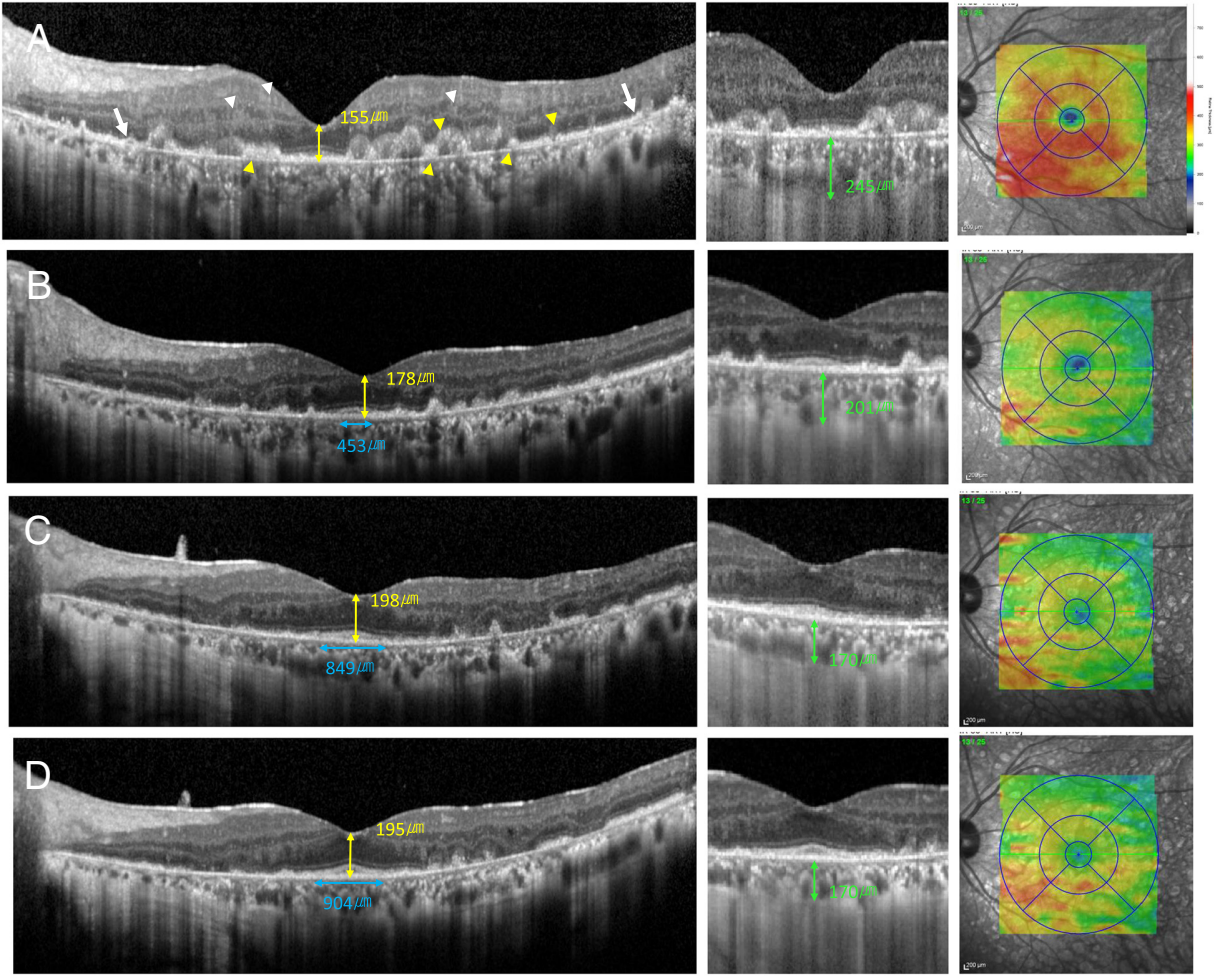
**Spectral-domain optical coherence tomography and enhanced depth imaging-OCT findings in the eye with PIOL.** The left column shows the SD-OCT images; the middle column, the EDI-OCT images; and the right column, the retinal thickness map. **(A)** Before treatment with methotrexate, **(B)** 1 month after initial methotrexate injection, **(C)** 6 months after initial injection, and **(D)** 9 months after initial injection. SD-OCT shows increased nodularity at the level of and above the retinal pigment epithelium (RPE) (yellow arrowheads), a separation of Bruch membrane from the RPE (arrows), damage of the RPE, disruption of the photoreceptor inner and outer segment (IS/OS) junction and external limiting membrane (ELM), multiple hyperreflective signals in the inner retina (white arrowheads), and foveal thinning (**A**, left). After initial injection of methotrexate, the hyperreflective infiltrations in the retina are markedly decreased, the RPE, the IS/OS junction, and ELM are restored, and the foveal thickness recovers (**B** to **D**, left). The subfoveal choroidal thickness gradually decreases in the EDI-OCT images (**A** to **D**, middle). A thickening of the retina is observed outside the central sector before treatment (**A**, right), but the retinal thickness gradually decreases outside the central sector after treatment (**B** to **D**, right).

SD-OCT was performed with the Heidelberg Spectralis (Heidelberg Engineering, Heidelberg, Germany). The foveal thickness, subfoveal choroidal thickness, and length of photoreceptor inner segment/outer segment (IS/OS) junction were manually measured using the caliper function. A retinal thickness map of the central 6.0-mm circle was made automatically by the built-in software. The SD-OCT images showed increased nodularity at the level of and above the RPE, a separation of Bruch membrane from the RPE, and a partial destruction of the RPE. There was also a disruption of the IS/OS junction, multiple hyperreflective signals in the inner retina, and foveal thinning (Figure 2A, left; Table 1).

After treatment, the degree of hyperreflective infiltration in the inner retina markedly decreased. The RPE and IS/OS junction were restored, and the foveal thickness recovered (Figure 2B,C,D, left; Table 1). Enhanced depth imaging OCT (EDI-OCT) showed that the subfoveal choroidal thickness gradually decreased during the treatment (Figure 2A,B,C,D, middle; Table 1).

Before treatment, the total macular volume in the central 6.0-mm circle was increased although the foveal thickness was reduced (Figure 2A, right; Table 1). After treatment, the total macular volume was reduced (Figures 2B,C,D, right; Table 1).

**Figure 3:**
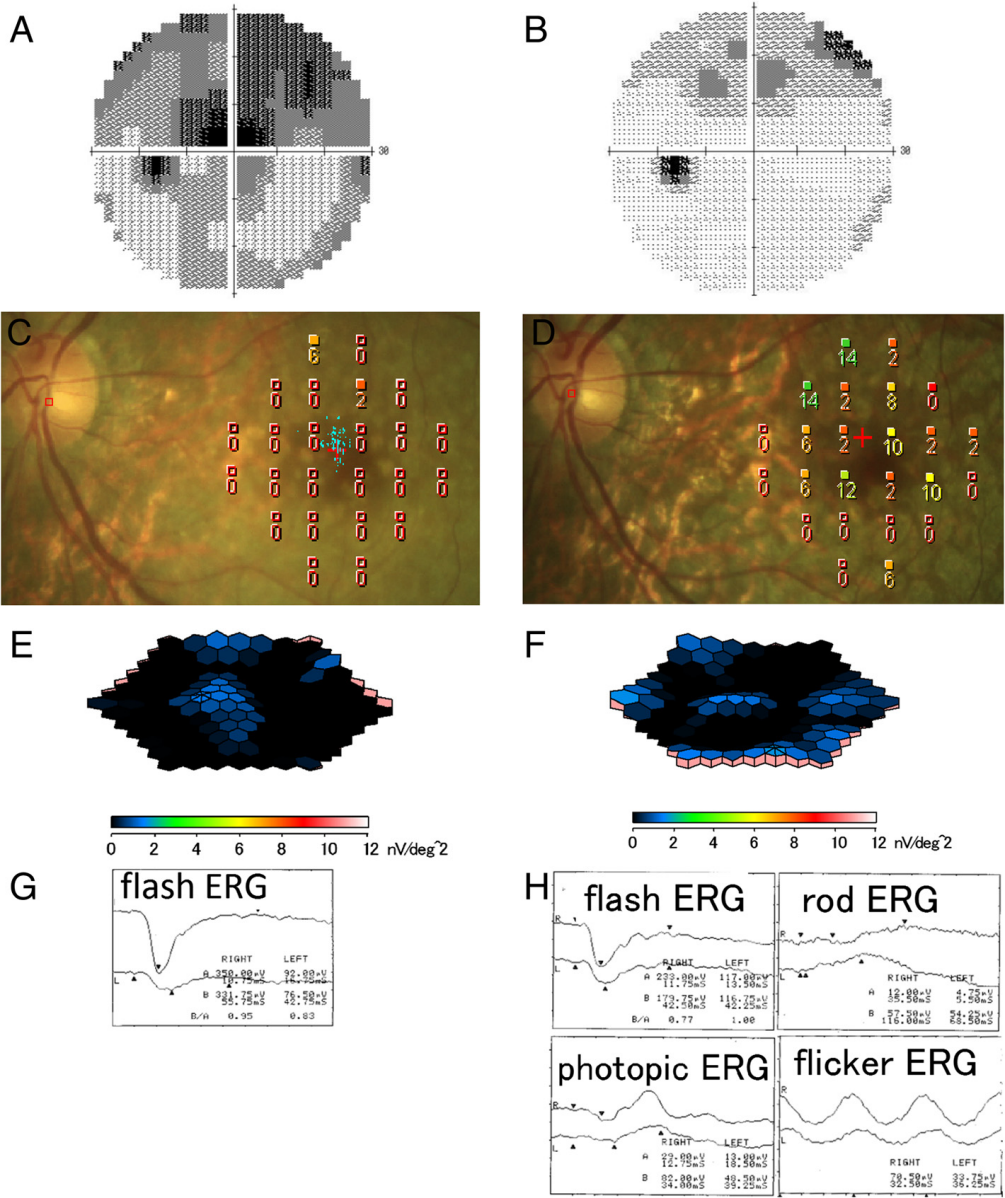
**Humphrey visual fields, microperimetry, multifocal electroretinogram (mfERGs), and full-field ERG findings with PIOL.** The left column shows images before methotrexate, and the right column shows images 9 months after treatment. **(A, B)** Humphrey visual field test, **(C, D)** microperimetry, **(E, F)** mfERG, and **(G, H)** full-field ERG. Mean deviation of 30-2 program in Humphrey Field Analyzer increases from -13.95 dB before treatment to -7.23 dB after treatment. Mean retinal sensitivity within the central 10° in microperimetry increases from 0.3 dB before treatment to 4.1 dB. Amplitudes of mfERGs are markedly decreased without improvement. ERGs are subnormal and do not improve after treatment. In addition, the rod ERG, photopic ERG, and 30-Hz flicker ERG are reduced.

Standard automated perimetry was performed with the Humphrey Field Analyzer (HFA, Carl Zeiss Meditec, Dublin, CA, USA), and the 30-2 program with the SITA was used. The mean deviation (MD) increased from -13.95 dB before treatment (Figure 3A) to -7.23 dB after treatment (Figure 3B).

The mean retinal sensitivity within the central 10° was measured with a fundus-related microperimeter (MP1, Nidek, Gamagori, Japan). The follow-up examinations were performed on the earlier tested retinal points. The Goldmann III stimuli and a 4-2 staircase strategy were used, and a rectangular 3° × 3° test grid with 24 stimulus locations covering an area of 10° was applied. The mean retinal sensitivity increased from 0.3 dB before treatment (Figure 3C) to 4.1 dB after treatment (Figure 3D).

Full-field ERGs (LE-4000, Tomey, Nagoya, Japan) and mfERGs (LE-4100, Tomey, Nagoya, Japan) were recorded according to the International Society for Electrophysiology of Vision (ISCEV) standards [7,8]. The amplitudes of the mfERGs were markedly decreased in the left eye before treatment (Figure 3E). During the follow-up period, the amplitudes of the mfERG remained markedly reduced (Figure 3F). The amplitudes of the scotopic, photopic, and 30-Hz flicker ERGs were reduced after the treatments (Figure 3G,H). Color vision with the Farnsworth D15 test showed tritan-like responses after the treatment.

### Discussion

Earlier, we found that the FAF images of the two eyes with PIOL had a granular pattern that consisted of hypoautofluorescent spots surrounded by hyperautofluorescent rings [6]. In addition, SD-OCT showed nodular hyperreflective areas not only under but also above the RPE. Because FAF depends on the distribution of lipofuscin in the RPE cells, it was suspected that the areas of lymphomatous infiltration into the sub-RPE space can alter the RPE metabolism leading to the hyperautofluorescence. Ishida et al. suggested that the hypoautofluorescence in PIOL patients is caused by RPE atrophy or the blockage of autofluorescence from the RPE by the tumor cells [5]. Casady et al. showed that the hyperautofluorescent spots on FAF changed to hypoautofluorescent spots corresponding to the atrophic areas in the RPE after treatment [4]. Similarly, we found that the FAF images had a granular pattern of slight hypoautofluorescence and hyperautofluorescence before treatment and patchy hypoautofluorescence corresponding to RPE atrophy after the treatments. These differences in the FAF findings may be useful for determining the sites of PIOL activity.

Fardeau et al. examined 244 patients with chronic uveitis who underwent vitreous sampling for cytological analysis [9]. They reported that the fovea in the OCT images was significantly thinner in the eyes with PIOL than in those with severe posterior uveitis. Also, our case had a reduction of foveal thickness before treatment.

Information about the visual fields in the eyes with PIOL is limited. Kim et al. reported that the visual fields were constricted in a case of PIOL and slightly recovered after treatment [10]. In our case, the visual field determined by HFA and microperimetry improved after treatment but not to the normal level. Yasuda et al. reported that the pre-treatment full-field ERGs were the negative type in the eyes with a PIOL suggesting that the inner retina was damaged more than the outer retina [11]. In their study, the ERG findings did not fully recover after chemotherapy [11]. In our case, the markedly decreased amplitudes of mfERGs and full-field ERGs also did not improve after treatment. Even though the RPE and IS/OS lines are restored and the foveal thickness on SD-OCT images are recovered, the global retinal damage due to the tumor infiltration into the retina may have persisted after successful treatment.

This study has limitations. We studied only one case of PIOL, and further studies examining a larger number of cases treated for PIOL will be needed to accurately determine the chronological changes of the ophthalmologic findings before and after treatment.

In conclusion, FAF and SD-OCT may be useful, noninvasive methods to evaluate the retina and choroid before and after treatment for PIOL. Regardless of the good visual acuity and improvement of retinal sensitivity, the amplitudes of the mfERGs remained markedly decreased. For better prognosis of retinal function, prompt treatment before lymphoma invasion of macular area may be necessary.

## Abbreviations

BCVA: best-corrected visual acuity; EDI-OCT: enhanced depth imaging OCT; FAF: fundus autofluorescence; HFA: Humphrey Field Analyzer; IL: interleukin; ISCEV: International Society for Electrophysiology of Vision; IS/OS: inner segment/outer segment; MD: mean deviation; mfERGs: multifocal electroretinograms; MTX: methotrexate; PCNSLs: primary central nervous system lymphomas; PIOL: primary intraocular lymphoma; RPE: retinal pigment epithelium; SD-OCT: spectral-domain optical coherence tomography.

## Competing interests

The authors do not have any proprietary interests in the materials described in the article.

## Authors’ contributions

ME managed the case and drafted the manuscript. YM supervised the management of the case, participated in the design of the study, and revised the manuscript critically. YH was involved in treating the case, reviewed the paper, and gave valuable comments. KS was involved in the medical care of the case and contributed to the collection of imaging data. TN was involved in performing surgery for diagnosis, reviewed the paper, and gave valuable comments. All authors read and approved the final manuscript.
